# The risk factors for chemotherapy myelosuppression in breast cancer: a systematic review and meta-analysis

**DOI:** 10.3389/fgene.2025.1704489

**Published:** 2025-12-08

**Authors:** Yuan yuan Zhu, Yang Li, De lan Zeng, Ni Li

**Affiliations:** 1 Department of Nursing College, Guangxi University of Traditional Chinese Medicine, Nanning, Guangxi, China; 2 Department of Operating Room, First Affiliated Hospital of Guangxi University of Traditional Chinese Medicine, Nanning, Guangxi, China

**Keywords:** breast cancer, chemotherapy-induced myelosuppression, risk factor, meta-analysis, incidence

## Abstract

**Aim:**

This systematic review and meta-analysis aimed to estimate the pooled prevalence and identify the risk factors associated with chemotherapy-induced myelosuppression (CIM) in breast cancer patients.

**Design:**

Systematic review and meta-analysis.

**Methods:**

PubMed, Cochrane Library, Embase, Web of Science, Medline, Scopus, CNKI, CBM,Wanfang Data Knowledge Service and VIP databases were searched from the inception to February 2025 for cross-sectional, cohort, and case-control studies investigating the prevalence and risk factors of CIM in breast cancer patients. Two researchers independently screened the literature according to the inclusion and exclusion criteria, and conducted data extraction and quality evaluation. Meta-analysis of the included literature was performed using Stata software.

**Results:**

A total of 24 studies involving 17,492 patients were included, and 21 potential risk factors were extracted. The pooled prevalence of CIM across all studies was 47.9%. Significant risk factors for CIM in breast cancer patients undergoing chemotherapy included Age, BMI, Pathological staging, hemoglobin, Lymphocyte, Diabetes, Liver function, history of radiotherapy, chemotherapy regimen, genetic factors, albumin. (all P < 0.05).

**Conclusion:**

CIM is a common and significant adverse effect in breast cancer patients undergoing chemotherapy, influenced by multiple risk factors. Identifying and understanding these factors can provide a crucial theoretical basis for healthcare professionals to develop targeted strategies for prevention and management, ultimately enhancing patient outcomes.

## 1 Introduction

Breast cancer (BC) is the most commonly diagnosed cancer among women and ranks second in cancer related mortality after lung cancer (Bray et al., 2024). According to data from the World Health Organization’s International Agency for Research on Cancer, approximately 1.7 million new cases of BC are diagnosed globally each year, accounting for 25% of all malignancies in women, with an estimated 521,900 deaths, representing 15% of female cancer related deaths (Loibl et al., 2021). In 2022, the global incidence of BC reached 2.3 million cases, with 670,000 deaths. These numbers are projected to rise by 38% and 68%, respectively, by 2050 (Ouyang and Shi, 2025; Kim et al., 2025).

Among the various treatment options for BC, chemotherapy remains one of the primary therapeutic modalities (Romeo et al., 2021). While chemotherapy is effective in controlling disease progression and improving survival, it is also associated with a range of adverse effects, including nausea, vomiting, appetite loss, weight loss, fatigue, and notably, myelosuppression (Lin et al., 2024). Chemotherapy-induced myelosuppression (CIM) is a serious treatment-related complication resulting from cytotoxic damage to hematopoietic stem and progenitor cells in the bone marrow. Clinically, CIM presents as anemia, thrombocytopenia, lymphocytopenia, and neutropenia, with an incidence rate as high as 40% (Goldschmidt et al., 2022; Hart et al., 2023). CIM not only compromises the efficacy of subsequent treatments but also adversely affects patient outcomes and quality of life, potentially leading to life-threatening consequences (Cann et al., 2025; Safaroghli-Azar et al., 2025). Therefore, identifying the prevalence and risk factors for CIM is of great clinical and public health significance for its prevention and management.

The risk factors associated with CIM have been extensively explored by researchers worldwide. Dr. Chun Chao’s study identified age, body mass index (BMI), TNM stage, and chemotherapy regimen as key factors (Chao et al., 2016). Susanna et al. further included baseline blood cell counts and comorbidities as additional risk variables (Hutajulu et al., 2023). While Liu Yang’s research introduced ethnicity as a potential factor (Liu et al., 2024a), notably, Liu Yang identified BMI as a significant risk factor, whereas Li’s study reported contrasting findings (Li et al., 2024). Such inconsistencies contribute to uncertainty and reduced reliability in clinical decision-making. While certain risk factors such as age, BMI, and pathological stage have been consistently validated, other variables including genetic predisposition, treatment cycles, baseline hematological parameters, surgical history, and performance status (e.g., Karnofsky Performance Status, KPS), remain controversial or insufficiently explored (Xia et al., 2024; Jiao et al., 2019; Moro et al., 2022; Ishikawa et al., 2021). Substantial heterogeneity exists across current studies regarding identified risk factors. Variability in study design, sample size, population characteristics, and statistical methodology has led to conflicting or inconclusive results. Given this fragmentation and inconsistency, reliance on individual studies alone is insufficient to fully elucidate the risk profile of CIM. Hence, there is a critical need to synthesize existing evidence through a systematic and quantitative approach.

Considerable heterogeneity exists among studies investigating risk factors for CIM in BC. Differences in study design, sample size, and analytical methods have produced inconsistent findings, limiting the reliability of current evidence. Systematic reviews and meta-analyses provide a rigorous and transparent framework for integrating results across studies to generate robust, evidence-based conclusions that can inform clinical decision-making (Stroup et al., 2000). Consequently, to address gaps in current evidence, this study conducts a systematic review and meta-analysis, aiming to provide a quantitative synthesis estimating the pooled prevalence and identifying key risk factors for CIM in BC patients, with the ultimate goal of informing clinical practice.

## 2 Methods

**FIGURE 1:**
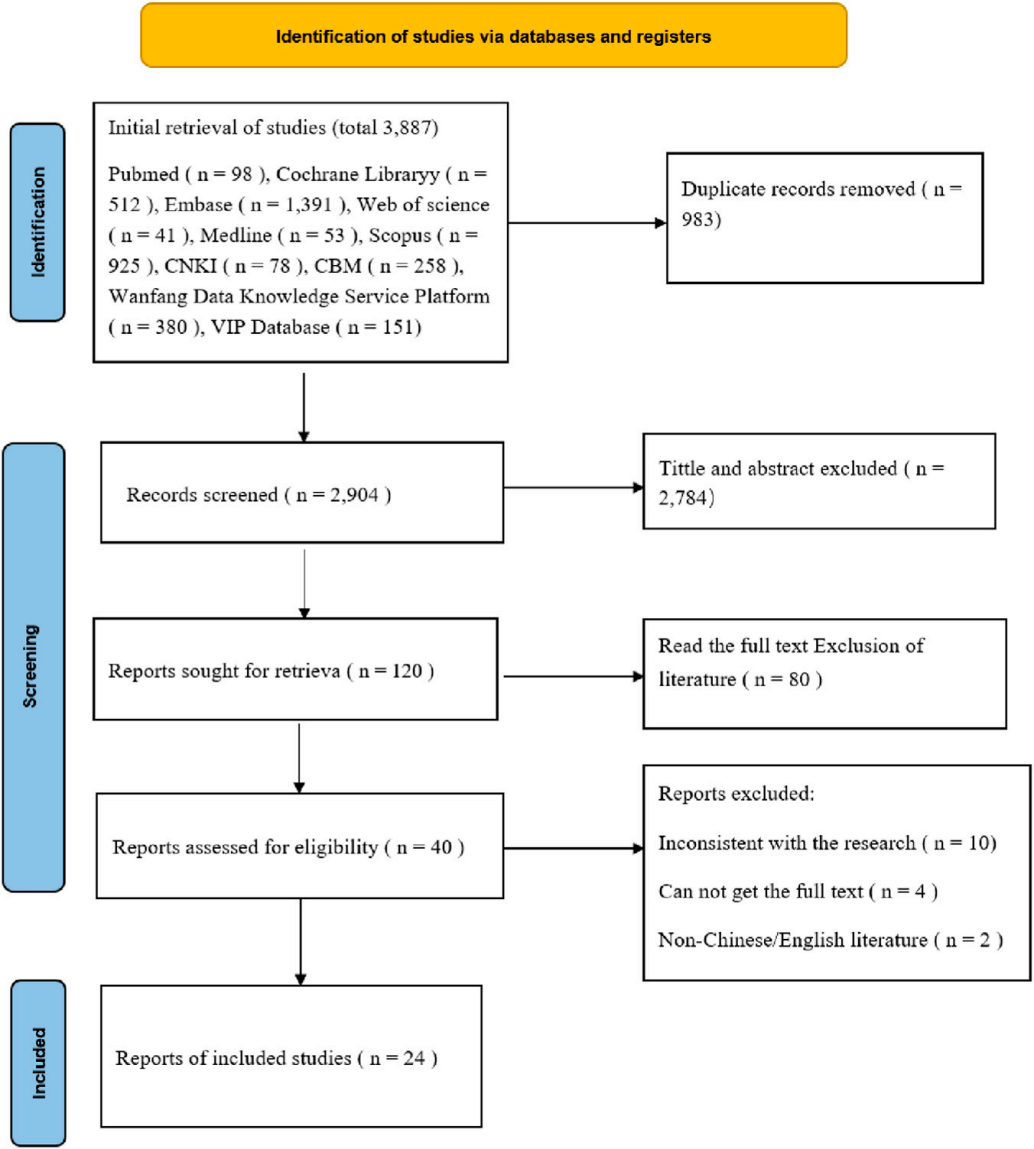
Preferred Reporting Items for Systematic Reviews and Meta-Analyses (PRISMA) flow diagram for the study selection process.

The meta-analysis was registered with PROSPERO (CRD420250655171) and reported using Preferred Reporting Items for Systematic Reviews and Meta-Analysis (PRISMA) guidelines (Figure 1).

### 2.1 Research strategy

We conducted a systematic literature search of PubMed, Cochrane Library, Embase, Web of Science, Medline, Scopus, CNKI, CBM, Wanfang Data Knowledge Service and VIP databases from inception to February 2025. Chinese search terms: “乳腺癌” OR “乳腺恶性肿瘤” OR “乳腺肿块” OR “乳腺癌”; “骨髓抑制/严重骨髓抑制/白细胞减少/中性粒细胞减少/贫血/血小板减少”. English search terms: Breast Neoplasms, Breast Tumor, Breast Cancer, Breast Malignant Tumor, Mammary Cancer, Breast Carcinoma, Breast Carcinomas; Bone marrow suppression/severe bone marrow suppression/leukopenia/neutropenia/anemia/thrombocytopenia. See supplementary materials for search strategy.

### 2.2 Criteria for study selection

Inclusion criteria:

① P (Population) target population: BC diagnosed by pathological tissue, age ≥18 years old;
② E (Exposure) exposure factors: factors that can affect the occurrence of bone marrow suppression in patients and exposure factors are clearly defined;
③ C (Control) control: no exposure factors;
④ O (Outcome) outcome indicators: the occurrence of bone marrow suppression. (Occurrence of CIM with a clearly stated definition and outcome indicators included neutropenia, lymphocytopenia, anemia, and ≥1 cytopenia in thrombocytopenia)

Excluded criteria:

① Different databases cross or duplicate published literature;
② Literature with incomplete information and incomplete data;
③ Repeated reports, case reports or review of the literature;
④ Literatures with incomplete statistical description or inappropriate statistical methods;
⑤ The original data did not provide literature that could be directly used or converted for this study, such as Odds Ratio (OR) or Relative Risk (RR) and 95% Confidence Interval (*CI*).

### 2.3 Data extraction

After the initial search, all results were imported into a reference management program (EndNote X9.3.2, Clarivate Analytics, Philadelphia, PA, USA). Two reviewers (Zhu and Li) screened the literature independently, extracted data, and cross-checked them according to the eligibility criteria. In case of disagreements, a third reviewer (Zeng) was involved in the discussion until a consensus was reached. The following data were extracted: author (year), study design, country/location, study design, sample size, and risk factors for CIM. The details of all the extracted data are shown in Table 1.

**TABLE 1:** Basic characteristics of the included studies and evaluation of methodological quality.

	Author (Year)	Country	Study design	Sample size	Prevalence	Risk factors	Literature quality evaluation
1	Zhang et al. (2010)	China	cross section study	219	27.85%	① ② ③ ⑲	6 AHRQ
2	Chan et al. (2012)	Singapore	cohort study	189	13.80%	②	7 NOS
3	Chaumard et al, (2012)	France	cross section study	378	64.00%	① ② ⑤ ⑯ ㉑	6 AHRQ
4	Altwairgi et al. (2013)	Canada	cohort study	239	21.00%	① ⑯	7 NOS
5	Wang et al. (2013)	China	cross section study	179	50.28%	① ⑯ ⑱	7 AHRQ
6	Cai et al. (2015)	China	cross section study	400	72.20%	① ② ⑤ ⑨ ⑩ ⑯	6 AHRQ
7	Chao et al. (2016)	United States of America	cross section study	11291	3.00%	① ⑤ ⑯	6 AHRQ
8	Fu et al. (2016)	China	cross section study	218	78.00%	② ⑥ ⑦	7 AHRQ
9	You et al. (2016)	China	case control study	190	50.00%	③ ⑭	8 NOS
10	Yuan et al. (2017)	China	cross section study	191	27.22%	② ③ ④ ⑤ ⑦ ⑧ ⑮ ⑯ ⑱	6 AHRQ
11	Sun et al. (2017)	China	cross section study	240	67.60%	① ⑤ ⑨ ⑩ ⑫ ⑬ ⑯	6 AHRQ
12	Fan et al. (2018)	China	cross section study	68	30.88%	⑰	6 AHRQ
13	Jiao et al. (2019)	China	cross section study	400	32.00%	② ⑤ ⑥ ⑲	7 AHRQ
14	He et al. (2019)	China	cross section study	583	56.26%	③ ⑤ ⑰ ㉑	6 AHRQ
15	Ishikawa et al. (2021)	Japan	cohort study	980	95.00%	① ② ③ ⑦ ⑪ ⑯	8 NOS
16	Kanbayashi et al. (2021)	Japan	cross section study	78	75.60%	③ ④ ⑮	8 AHRQ
17	Kimura et al. (2021)	Japan	cross section study	196	35.71%	① ⑪ ⑫ ⑬	6 AHRQ
18	Moro et al. (2022)	Japan	cross section study	61	27.86%	③ ⑪	8 AHRQ
19	Wang et al. (2022)	China	cross section study	243	27.98%	⑪ ⑭	6 AHRQ
20	Hutajulu et al. (2023)	Indonesia	cross section study	180	78.00%	① ② ③ ④ ⑤ ⑥ ⑦ ⑨ ⑩ ⑯ ⑳	7 AHRQ
21	Huang et al. (2023)	China	case control study	207	51.00%	① ② ③ ⑧ ⑩ ⑳	8 NOS
22	Cao et al. (2023)	China	cross section study	183	67.21%	① ④ ⑦ ⑭ ⑯ ㉑	6 AHRQ
23	Li et al. (2024)	China	cross section study	270	46.29%	① ⑤ ⑥ ⑮ ㉑	7 AHRQ
24	Liu et al. (2024a)	China	case control study	309	47.25%	① ② ③ ⑥ ⑦ ⑧ ⑪ ⑮ ⑰ ⑳	8 NOS

AHRQ, agency for healthcare research and quality; NOS, Newcastle-Ottawa Scale.

Influencing factor: ①Age; ②BMI; ③Pathological staging; ④Molecular typing; ⑤Hemoglobin; ⑥White blood cell; ⑦Neutrophils; ⑧Iymphocyte; ⑨Hypertension; ⑩Diabetes; ⑪Prophylactic G-CSF; ⑫Liver function; ⑬Renal function; ⑭History of radiotherapy; ⑮Platelet count; ⑯Chemotherapy regimen; ⑰Chemotherapy cycles; ⑱KPS; ⑲Genetic factors; ⑳Albumin; ㉑Genetic factors.

### 2.4 Quality assessment

Two researchers (Zhu and Li) independently assessed the quality of all the observational studies. For the cohort and case-control studies, the Newcastle-Ottawa Scale (NOS) (Wells et al., 2013) was applied to evaluate each study’s selection, comparability, and outcome/exposure. The scale score varied from 0 to 9 points. Studies with scores of ≥7 points were considered to have a low risk; 4-6 points, moderate risk; and <3 points, high risk. For the cross-sectional studies, the Agency for Healthcare Research and Quality (AHRQ) (Hartling et al., 2012) tool was used to assess the risk of bias by answering the 11-item questions with “Yes”, “No, “and”, “Unclear”, respectively, where “Yes” is 1 point and “No or Unclear” is 0 point. A score of 0–3 is low quality, 4 - 7 is medium quality, and 8–11 is high quality (Zeng et al., 2012). The two authors discussed together and turned to the third author for any discrepancies. After literature quality evaluation, only medium and high quality literatures were included for meta analysis.

### 2.5 Data analysis

We performed a meta-analysis to estimate the incidence and risk factors of CIM in BC. Given the variability among studies, a random-effects model was applied to calculate pooled odds ratios (ORs) and 95% confidence intervals (CIs). Heterogeneity was assessed using Cochran’s Q test and the I^2^ statistic, and logit transformation was applied to normalize non-normally distributed incidence data.

Considering the cross-country variations in study design, patient age, and chemotherapy regimens, substantial heterogeneity was expected. To identify its potential sources and ensure a hypothesis-driven analysis, we conducted subgroup and meta-regression analyses according to region, age, and treatment regimen.

For the analysis of risk factors, adjusted ORs, relative risks (RRs), and hazard ratios (HRs) extracted from the original studies were combined to obtain pooled effect estimates. HRs and ORs/RRs were synthesized separately to maintain analytical consistency.

Publication bias was assessed when at least 10 studies were available. The potential for publication bias was assessed by inspecting funnel plots and using Egger’s test. If potential publication bias was detected, we conducted trim and fill analysis with contour-enhanced funnel plots. Data analysis was conducted with Stata 17.0, *P* < 0.05 was considered statistically significant.

## 3 Results

### 3.1 Search results

A total of 3,887 articles were retrieved, and after preliminary screening, 983 duplicate articles were excluded. Subsequently, 2,784 articles were further excluded by reading the title and abstract. By reading the full text, 96 articles were excluded, 24 articles were finally included. Including 16 Chinese articles and 8 English articles (Chao et al., 2016; Hutajulu et al., 2023; Liu et al., 2024a; Li et al., 2024; Jiao et al., 2019; Moro et al., 2022; Ishikawa et al., 2021; Sun et al., 2017; Cai et al., 2015; Zhang et al., 2010; Fan et al., 2018; He et al., 2019; Yuan et al., 2017; You et al., 2016; Wang et al., 2013; Wang et al., 2022; Huang et al., 2023; Fu et al., 2016; Kimura et al., 2021; Chaumard et al., 2012; Chan et al., 2012; Cao et al., 2023; Kanbayashi et al., 2021; Altwairgi et al., 2013) reviews met the inclusion criteria and were incorporated in the meta-analysis (Figure 1).

### 3.2 Characteristics of included studies

The characteristics of the 24 studies are described in Table 1. 16 (Liu et al., 2024a; Li et al., 2024; Xia et al., 2024; Jiao et al., 2019; Sun et al., 2017; Cai et al., 2015; Zhang et al., 2010; Fan et al., 2018; He et al., 2019; Yuan et al., 2017; You et al., 2016; Wang et al., 2013; Wang et al., 2022; Huang et al., 2023; Fu et al., 2016; Cao et al., 2023) were from China, four (Moro et al., 2022; Ishikawa et al., 2021; Kimura et al., 2021; Kanbayashi et al., 2021) were from Japan, One each was from France (Chaumard et al., 2012), Canada (Altwairgi et al., 2013), United States (Chao et al., 2016), and Indonesia (Hutajulu et al., 2023). There were three cohort studies, three case-control studies, 18 cross-sectional studies.

### 3.3 Prevalence of chemotherapy-induced myelosuppression

**FIGURE 2:**
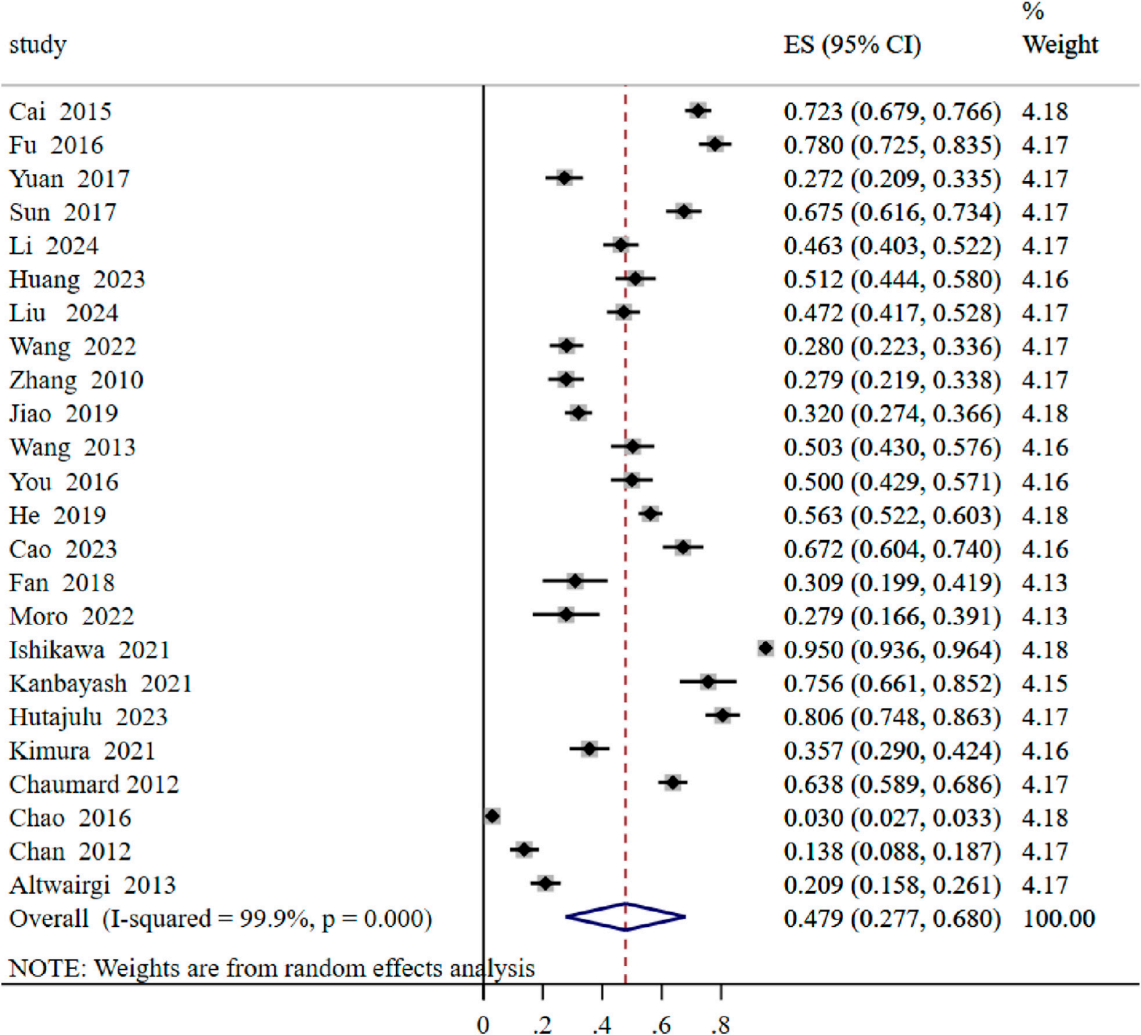
Prevalence of chemotherapy-induced myelosuppression.

In the 24 studies available for the meta-analysis, the prevalence of CIM ranged from 3.00% to 78.00%. Based on a random-effects model-based meta-analysis conducted on all data points, over-all CIM prevalence was estimated to be 47.9% (95% *CI*: 0.277–0.680,I^2^ = 99.9%, *P* < 0.001) (Figure 2).

**FIGURE 3:**
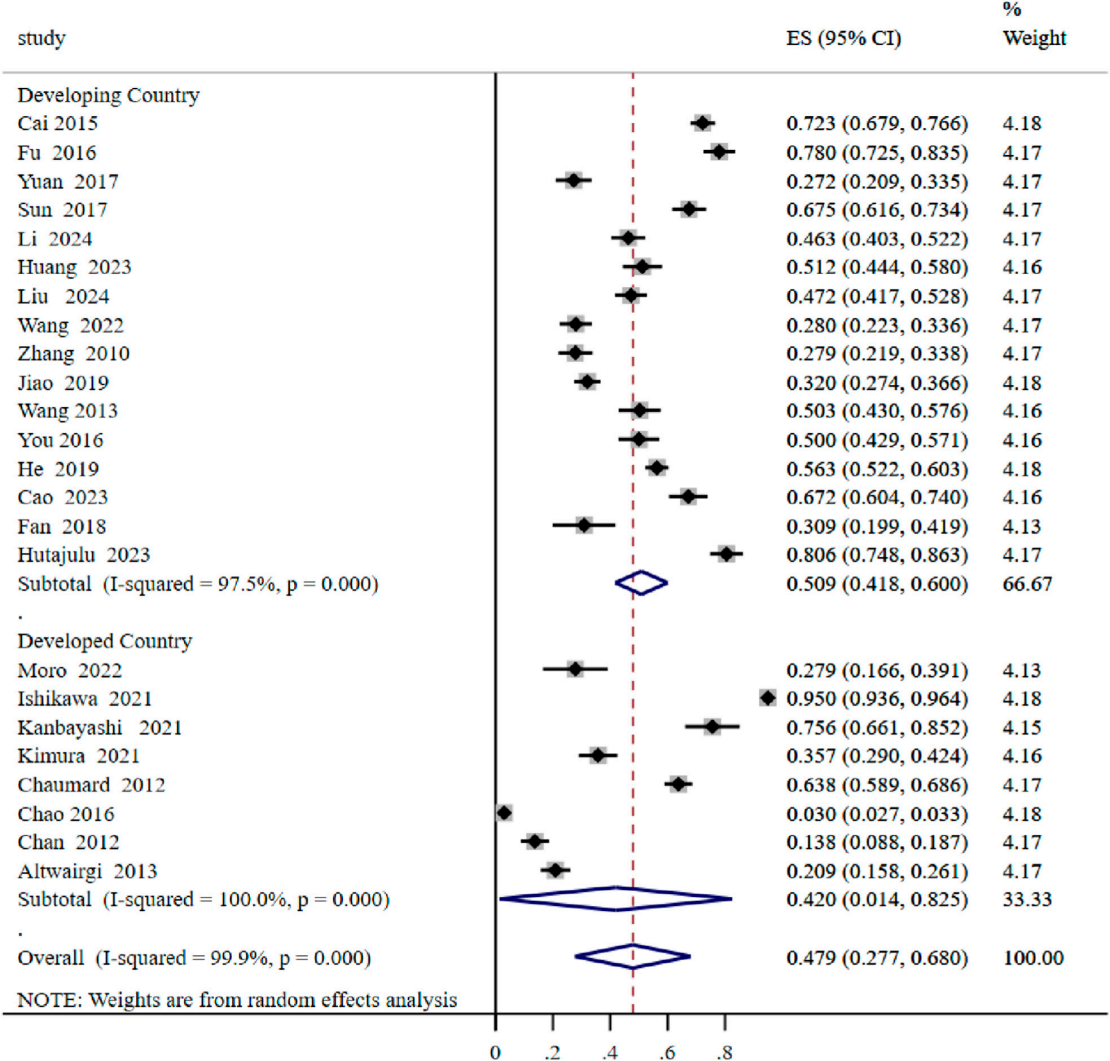
Subgroup analysis of rat.

Further subgroup analysis revealed a significant difference in the prevalence of CIM between developing and developed countries. The prevalence in developing countries was notably higher, at 50.9%, compared to 42.0% in developed countries (Figure 3).

### 3.4 Meta-analysis results

This study included a total of 24 studies encompassing 17,492 patients. Meta-analysis of 21 influencing factors showed that Age, BMI, Pathological stage, Hemoglobin, Lymphocyte, Diabetes, Liver function, Radiotherapy history, Chemotherapy regimen, Gene factors and Albumin were 11 Risk factors for CIM. Combined OR value and 95% CI were statistically significant (*P* < 0.05) (Table 2).

**TABLE 2:** Meta-analysis results of risk factors for chemotherapy-induced myelosuppression.

lnfluencing factor	Eligible studies	Heterogeneity test		Effects models	95 %*CI*	OR	Z	*P*
		*I* ^2^ (%)	*P*					
Age	16 (Chao et al., 2016; Hutajulu et al., 2023; Liu et al., 2024a; Ishikawa et al., 2021; Sun et al., 2017; Cai et al., 2015; Zhang et al., 2010; Yuan et al., 2017; Wang et al., 2013; Huang et al., 2023;Kimura et al., 2021; Chaumard et al., 2012; Cao et al., 2023; Altwairgi et al., 2013)	74.5%	<0.001	random	1.16-2.21	1.60	2.88	0.004
BMI	14 (Chao et al., 2016; Hutajulu et al., 2023; Liu et al., 2024a; Jiao et al., 2019; Cai et al., 2015; Zhang et al., 2010; Yuan et al., 2017; Huang et al., 2023; Fu et al., 2016; Chaumard et al., 2012; Chan et al., 2012)	74.5%	<0.001	random	1.08-2.22	1.55	2.37	0.018
Pathological staging	10 (Hutajulu et al., 2023; Liu et al., 2024a; Moro et al., 2022; Ishikawa et al., 2021; Zhang et al., 2010; He et al., 2019; Yuan et al., 2017; You et al., 2016; Huang et al., 2023)	80.1%	<0.001	random	1.28-3.18	2.02	3.02	0.003
Molecular typing	7 (Moro et al., 2022; Yuan et al., 2017; Cao et al., 2023)	0.0%	0.754	fixed	0.74-1.21	0.95	0.44	0.662
Hemoglobin	9 (Chao et al., 2016; Hutajulu et al., 2023; Li et al., 2024; Jiao et al., 2019; Sun et al., 2017; Cai et al., 2015; He et al., 2019; Chaumard et al., 2012)	96.3%	<0.001	random	1.06-4.39	2.16	2.12	0.034
White blood cell	5 (Hutajulu et al., 2023; Liu et al., 2024a; Li et al., 2024; Jiao et al., 2019; Huang et al., 2023; Fu et al., 2016)	53.6%	0.071	random	0.92-3.55	1.80	1.71	0.087
Neutrophils	6 (Hutajulu et al., 2023; Liu et al., 2024a; Ishikawa et al., 2021; Yuan et al., 2017; Fu et al., 2016)	39.8%	0.140	fixed	0.69-0.96	0.82	2.42	0.015
Iymphocyte	3 (Liu et al., 2024a; Huang et al., 2023; Kanbayashi et al., 2021)	77.3%	0.012	random	1.55-4.23	2.56	3.66	0.000
Hypertension	4 (Hutajulu et al., 2023; Wells et al., 2013; Hartling et al., 2012; Zhang et al., 2010)	32.9%	0.225	fixed	0.35-1.26	0.67	1.25	0.210
Diabetes	4 (Hutajulu et al., 2023; Liu et al., 2024a; Cai et al., 2015; Huang et al., 2023)	53.1%	0.094	fixed	1.08-278	1.73	2.26	0.024
Prophylactic G-CSF	5 (Liu et al., 2024a; Moro et al., 2022; Ishikawa et al., 2021; Wang et al., 2022; Kimura et al., 2021)	93.1%	<0.001	random	0.04-7.64	0.58	0.42	0.676
Liver function	2 (Sun et al., 2017; Kimura et al., 2021)	0.0%	0.094	fixed	2.04-6.39	3.62	4.42	0.000
Renal function	2 (Sun et al., 2017; Kimura et al., 2021)	0.0%	0.827	fixed	0.37-1.53	0.75	0.78	0.435
History of radiotherapy	3 (You et al., 2016; Wang et al., 2022; Cao et al., 2023)	0.0%	0.584	fixed	3.46-17.28	7.74	4.99	0.000
Platelet count	4 (Liu et al., 2024a; Li et al., 2024; Yuan et al., 2017; Kanbayashi et al., 2021)	11.6%	0.355	fixed	1.00-1.00	1.00	0.01	0.994
Chemotherapy regimen	20 (Chao et al., 2016; Hutajulu et al., 2023; Sun et al., 2017; Cai et al., 2015; Yuan et al., 2017; Chaumard et al., 2012; Cao et al., 2023; Altwairgi et al., 2013)	87.6%	<0.001	random	1.57-3.68	2.40	4.02	0.000
Chemotherapy cycles	6 (Liu et al., 2024a; Fan et al., 2018; He et al., 2019; Wang et al., 2022)	85.4%	<0.001	random	0.39-3.24	1.13	0.23	0.821
KPS	2 (Yuan et al., 2017; Wang et al., 2013)	97.8%	<0.001	random	0.08-1268.17	10.06	0.94	0.349
Genetic factors	3 (Jiao et al., 2019; Fu et al., 2016)	0.0%	0.690	fixed	2.24-9.29	4.56	4.18	0.000
Albumin	3 (Hutajulu et al., 2023; Liu et al., 2024a; Huang et al., 2023)	0.0%	0.897	fixed	1.53-3.99	2.47	3.71	0.000
History of surgery	4 (Li et al., 2024; He et al., 2019; Chaumard et al., 2012; Cao et al., 2023)	85.0%	<0.001	random	0.35-3.02	1.03	0.05	0.958

### 3.5 Publication bias and sensitivity analysis

After Meta-analysis, the results showed that: Age (OR = 1.60, I^2^ = 74.5%,95% *CI*: 1.16–2.21); BMI (OR = 1.55, I^2^ = 74.5%,95% *CI*: 1.08–2.22); Pathological staging (OR = 2.02, I^2^ = 80.1%,95% *CI*: 1.28-3.18); Chemotherapy regimen (OR = 2.40, I^2^ = 87.6%, 95% *CI*:1.57–3.68). Heterogeneity is high, subgroup analysis was performed to explore the source of heterogeneity.

**FIGURE 4:**
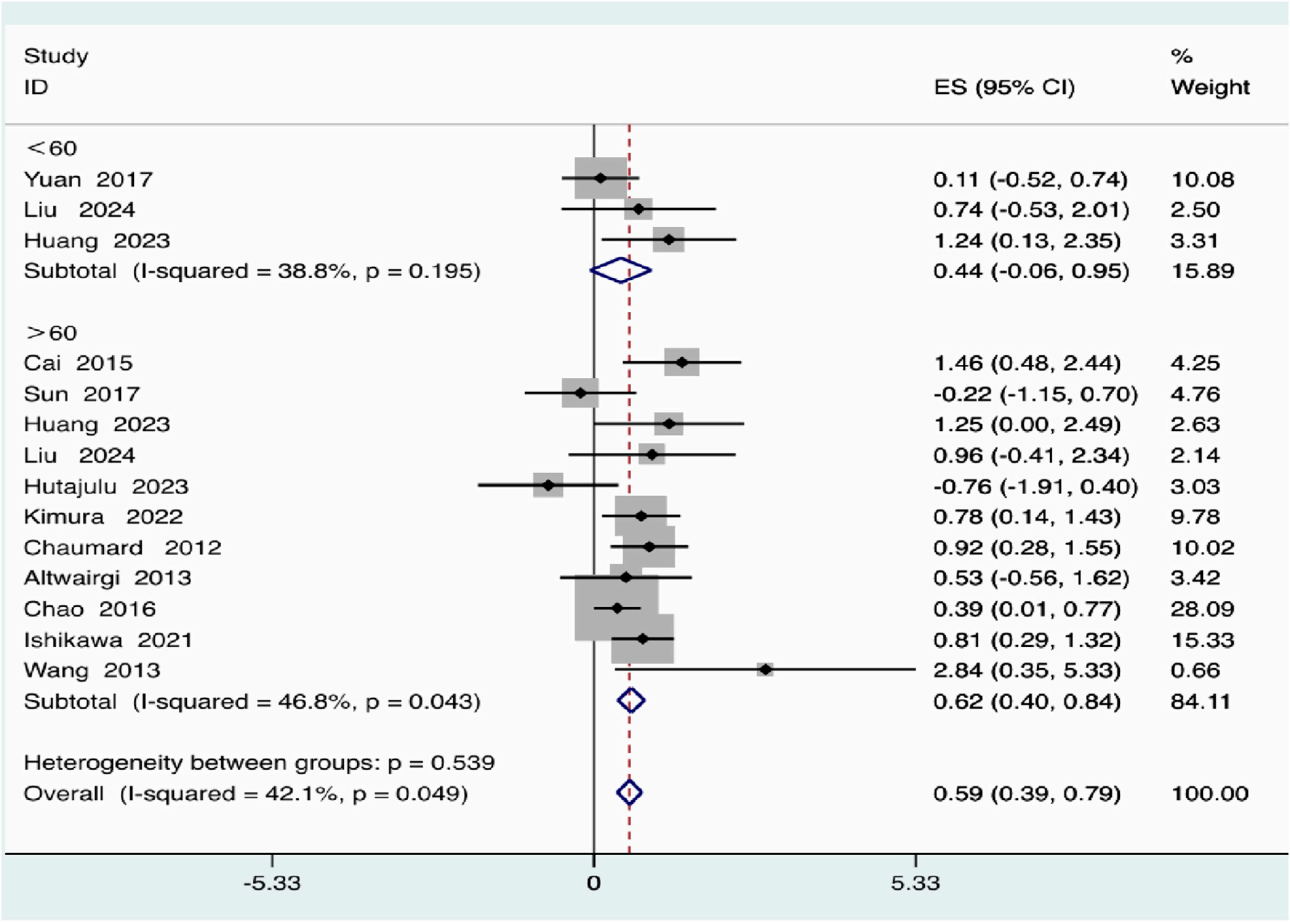
Age subgroup analysis.

Regarding Age, individuals over 60 years exhibited a significantly higher risk (effect size = 0.62, 95% *CI*: 0.40–0.84) compared to those under 60 years (effect size = 0.44, 95% *CI*: −0.06–0.95), although the difference between age groups was not statistically significant *(P* = 0.539) (Figure 4).

**FIGURE 5:**
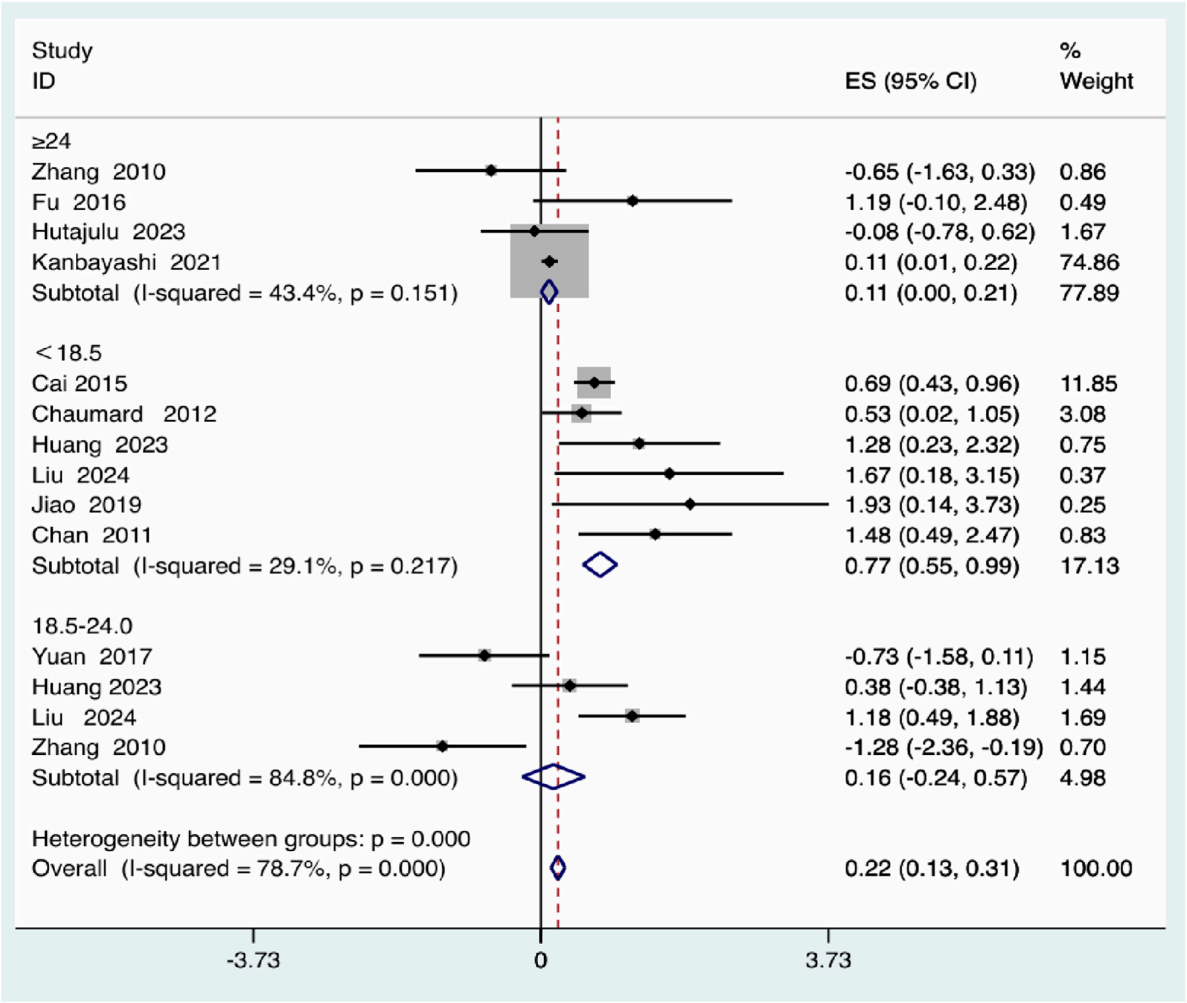
BMI subgroup analysis.

For BMI, patients with a BMI <18.5 demonstrated a significantly elevated risk (effect size = 0.77, 95% *CI*: 0.55–0.99), whereas those with a BMI ≥24 exhibited a much smaller increase (effect size = 0.11, 95% *CI*: 0.00–0.21). The BMI 18.5–24.0 group showed no statistically significant effect and high heterogeneity (I^2^ = 84.8%), with significant between-group differences (*P* = 0.000) (Figure 5).

**FIGURE 6:**
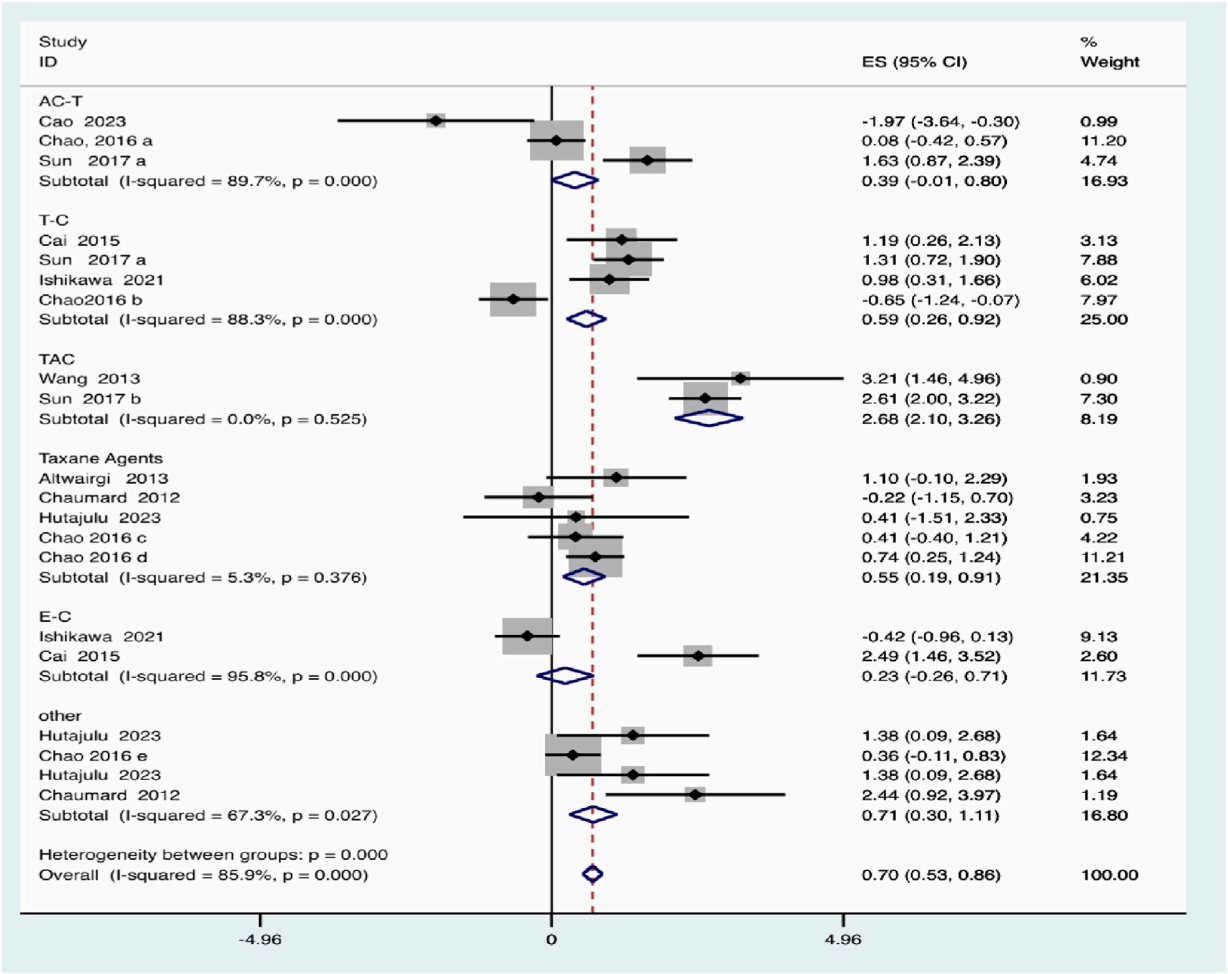
Chemotherapy regimen subgroup analysis.

For the Chemotherapy regimen, the TAC (Taxanes + Adriamycin + Cyclophosphamide) regimen was associated with the highest effect size (2.68, 95% *CI*: 2.10–3.26) and no heterogeneity, indicating a consistent and marked risk increase. Taxane agents and T-C (Taxanes-Cyclophosphamide) regimens also demonstrated elevated risks (0.55 and 0.59, respectively), albeit with varying degrees of heterogeneity. In contrast, AC-T (Anthracyclines + Cyclophosphamide-Taxanes) and E-C (Epirubicin-Cyclophosphamide) regimens did not show statistically significant effects and were characterized by substantial heterogeneity. Between-group heterogeneity was significant (*P* = 0.000), underscoring the role of regimen type as a major source of variability (Figure 6).

**FIGURE 7:**
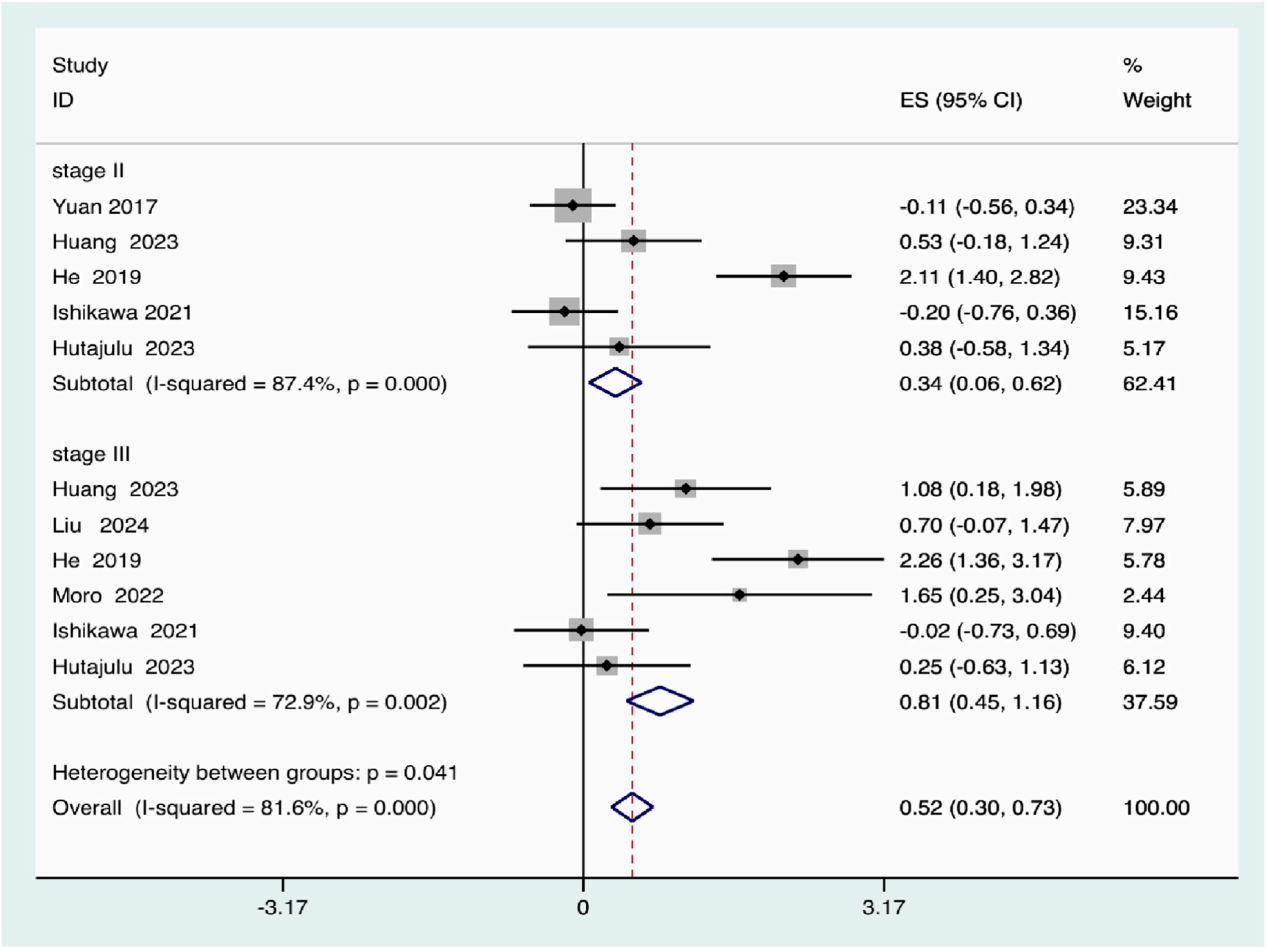
Pathological staging subgroup analysis.

For Pathological staging, Stage II patients showed a modest but significant increase in risk (effect size = 0.34, 95% *CI*: 0.06–0.62), while Stage III patients exhibited a higher and statistically significant risk (effect size = 0.81, 95% *CI*: 0.45–1.16), with significant differences between stages (*P* = 0.041) (Figure 7).

## 4 Discussion

Twenty-one studies were included in the present meta - analysis, which involved 17,492 subjects; the pooled prevalence of CIM was 47.9%. Additionally, the study’s findings revealed that developing countries (50.9%), have a higher incidence of CIM than developed countries (42.0%). Out of the 21 potential influencing factors examined, 11 were identified as statistically significant risk factors associated with the condition. The risk factors include Age, BMI, Pathological staging, Hemoglobin, Lymphocyte, Diabetes, Liver function, History of radiotherapy, Chemotherapy regimen, Genetic factors, and Albumin.

### 4.1 Demographic and physiological factors

Age is an important demographic predictor of CIM, as it comprehensively reflects a patient’s physiological reserve and metabolic capacity. Research outcomes indicate that elderly patients (particularly those aged ≥60 years) are significantly more likely to develop severe bone marrow suppression following cytotoxic chemotherapy compared to younger individuals,which is consistent with many research results (Liu et al., 2024a; Li et al., 2024; Du et al., 2025).

This increased risk is primarily attributed to age-related deterioration of the bone marrow microenvironment, reduced numbers and functionality of hematopoietic stem cells, and diminished regenerative capacity for white blood cells, red blood cells, and platelets (Wang L. et al., 2023). Additionally, age-associated decline in Liver and kidney reserve function can delay the clearance of chemotherapeutic agents, resulting in elevated plasma drug concentrations and exacerbated bone marrow toxicity (Crawford et al., 2024). Chronic low-grade inflammation and oxidative stress, along with impaired DNA damage repair mechanisms common in the elderly, further compromise the bone marrow niche and hematopoietic stem cell function, increasing susceptibility to CIM (Wang et al., 2024).

BMI also serves as a critical indicator of nutritional status, metabolic function, and demonstrates a bidirectional relationship with CIM risk. In Kong’s analysis of patients (Kong et al., 2025), underweight status was identified as a potential risk factor for overall survival compared with normal weight (HR 1.15, 95% *CI*: 0.98–1.35; *P* = 0.08). Low BMI often reflects malnutrition and micronutrient deficiencies (e.g., iron, folate, vitamin *B*12), which are essential for hematopoiesis and may hinder bone marrow recovery and regeneration (Wang Q. et al., 2023; Zhao et al., 2025). However, high BMI does not necessarily confer protection against CIM. For lipophilic drugs such as paclitaxel, accumulation in adipose tissue can lead to delayed clearance and prolonged bone marrow exposure to cytotoxic agents (Elwood et al., 2018). Moreover, some studies have described an “obesity paradox,” suggesting that adipose tissue may buffer peak plasma concentrations of chemotherapy agents and, in certain contexts, exert a protective effect against toxicity (Petrelli et al., 2021).

### 4.2 Physiological indicators are the influencing factors of CIM

The analysis revealed that patients with low pre-chemotherapy hemoglobin, Lymphocyte and albumin levels had a significantly increased risk of developing CIM, a finding consistent with results from multiple clinical studies (Li et al., 2024; Wang et al., 2021; Su et al., 2021). Anemia not only indicates a decline in hematopoietic system reserve capacity but may also reflect a combination of chronic inflammation, malnutrition, and tumor-induced bone marrow infiltration. These conditions collectively reduce the body’s tolerance to chemotherapy and heighten the risk of bone marrow toxicity (Brigle et al., 2017).

Lymphocytes play a pivotal and complex role in CIM. Although chemotherapy directly suppresses their proliferation and induces apoptosis leading to a marked reduction in peripheral counts these immune cells still contribute substantially to immune surveillance, immune reconstitution, and regulation of the bone marrow microenvironment (Liu et al., 2024b; Kenswil et al., 2022). In particular, T cells support immune surveillance and modulate immune responses, helping to control secondary infections and tumor relapse after chemotherapy. Even though the immune system is suppressed during treatment, recovery of these immune cells after therapy facilitates restoration of immune function, reduces infection rates, and improves clinical outcomes (Hong et al., 2024). Therefore, Lymphocytes not only reflect the status of the immune system during CIM but are also closely linked to clinical outcomes and immune recovery.

Mechanistically, anemia places the body’s erythropoietic system under compensatory stress, which consumes bone marrow resources and impairs the production of other hematopoietic lineages such as white blood cells and platelets. In addition, tumour-related chronic inflammation can disrupt iron metabolism and alter erythropoietin (EPO) function (Su et al., 2021; Brigle et al., 2017), further exacerbating the cycle of anemia and bone marrow dysfunction. Moreover, anemia often coexists with hypoalbuminemia and impaired nutritional absorption, which further compromises the patient’s overall tolerance to chemotherapy.

### 4.3 Comorbidities and disease status

Among BC patients undergoing cytotoxic chemotherapy, CIM is typically more prevalent in those with diabetes (Hutajulu et al., 2023). Diabetes is associated with persistent low-grade inflammation, characterized by elevated levels of proinflammatory cytokines such as IL-1β, IL-6, and TNF-α. These cytokines disrupt the bone marrow microenvironment, inhibit hematopoietic stem cell proliferation and differentiation, and impair the production of white blood cells, red blood cells, and platelets (Jiang et al., 2025; Pignalosa et al., 2021). Moreover, diabetes-related microangiopathy leads to bone marrow hypoperfusion and impaired microcirculation, creating a state of chronic hypoxia and nutrient deficiency in hematopoietic tissue, thereby increasing susceptibility to chemotherapy-induced toxicity (Fadini and Albiero, 2022).

The Liver plays a critical role in the metabolism and clearance of many commonly used chemotherapeutic agents. The results of this study showed that the risk of CIM in patients with impaired Liver function was 1.7 times higher than that in normal patients. Drugs such as taxanes (e.g., docetaxel, paclitaxel), anthracyclines (e.g., doxorubicin), and vinca alkaloids (e.g., vincristine) are primarily metabolized and excreted through hepatic and biliary pathways. Impaired Liver function can delay drug clearance, leading to prolonged systemic exposure and elevated plasma concentrations, thus exacerbating bone marrow suppression (Field et al., 2008). In addition, patients with hepatic dysfunction often present with nutritional deficiencies, hypoalbuminemia, and impaired coagulation (Ricart, 2017).

This study also identified tumor pathological stage as a significant factor influencing the risk of CIM. Patients with advanced-stage cancer (particularly stage III and IV) exhibit a significantly higher incidence of CIM compared to those with early-stage disease, a finding supported by several prior studies (Moro et al., 2022). Which impose greater myelosuppressive burdens; second, advanced tumors are often accompanied by systemic deterioration, including malnutrition, systemic inflammatory response (elevated CRP), and organ dysfunction, all of which impair tolerance to chemotherapy toxicity (Lalami and Klastersky, 2017; Hagiwara et al., 2022). This phenomenon is particularly pronounced in patients with advanced solid tumors such as gastric, lung, and BC, where stage III/IV disease is frequently associated with higher rates of neutropenia and thrombocytopenia (Wang et al., 2021).

### 4.4 Treatment-related factors

A history of radiotherapy and the specific chemotherapy regimen employed are important risk factors for CIM. Additionally, patients with prior radiotherapy experience a significantly increased risk of CIM, particularly neutropenia and thrombocytopenia-consistent with findings from several large-scale clinical studies (Wang et al., 2016; Upadhyay et al., 2021). Radiotherapy exerts a cumulative toxic effect on the hematopoietic system. Anatomical regions such as the pelvis, lumbar spine, and femur contain abundant active red bone marrow and serve as primary sites of hematopoiesis. Localized radiation in these areas can cause structural damage to the bone marrow stroma, induce apoptosis of mesenchymal stem cells, disrupt vascular integrity, and promote inflammatory fibrosis all of which markedly impair hematopoietic function at the irradiated sites (Majeed et al., 2023). Even months or years following radiotherapy, regenerative capacity in these areas may remain incomplete, rendering patients more vulnerable to severe bone marrow suppression during subsequent chemotherapy (Mohanti and Bansal, 2005). Furthermore, when the interval between radiotherapy and chemotherapy is short (e.g., in concurrent chemoradiotherapy or sequential chemotherapy initiated within 4 weeks), the bone marrow may not have sufficient time to recover before being subjected to another cytotoxic insult. This overlapping of treatment cycles can intensify the severity of CIM.

In this study, subgroup analyses based on chemotherapy regimens revealed notable differences in the risk of myelosuppression. Among the evaluated protocols, the TAC regimen (Taxanes + Adriamycin + Cyclophosphamide) showed the highest pooled risk, followed by taxane-based and T-C (Taxanes + Cyclophosphamide) regimens. In contrast, AC-T (Anthracyclines + Cyclophosphamide - Taxanes) and E-C (Epirubicin + Cyclophosphamide) regimens demonstrated lower or nonsignificant risks with greater heterogeneity. These findings suggest that taxane- and anthracycline-containing regimens, especially when combined with cyclophosphamide, contribute substantially to hematologic toxicity.

In addition, although most included studies did not stratify myelosuppression by hematologic type or toxicity grade, our meta-analysis still reflects the overall hematologic burden of chemotherapy. Future studies should adopt standardized grading systems (e.g., CTCAE Grades I–IV) and classify myelosuppression by type such as neutropenia, anemia, and thrombocytopenia to improve understanding of the clinical severity and frequency of hematologic toxicities and enhance the interpretability of meta-analytic findings.

The choice of chemotherapy regimen is another critical determinant of CIM risk. Our analysis revealed that combination chemotherapy, dose-dense protocols, and regimens containing highly myelotoxic agents were significantly associated with a higher incidence of CIM. Combination therapy enhances tumor cell cytotoxicity through synergistic mechanisms but simultaneously increases hematopoietic suppression. For instance, regimens such as AC-T (doxorubicin and cyclophosphamide followed by paclitaxel) have been reported to exert marked myelotoxic effects, with paclitaxel particularly impairing bone marrow recovery (Liu et al., 2024a).

According to the National Comprehensive Cancer Network (NCCN) Clinical Practice Guidelines in Oncology, the risk of febrile neutropenia (FN) following prior chemotherapy and radiotherapy ranges from 10% to 20% (Crawford et al., 2017).

### 4.5 Genetic factors

With the ongoing advancement of pharmacogenomics, increasing attention has been directed toward the influence of individual genetic variations on the metabolism and toxicity of chemotherapeutic agents. This study further supports previous findings that polymorphisms in genes encoding drug-metabolizing enzymes and drug transporters are significantly associated with the risk of chemotherapy induced myelosuppression CIM (Sharma et al., 2021). These results highlight the potential clinical value of genetic screening in the personalized management of chemotherapy.

Genes encoding ATP-binding cassette transporters, for example, play a critical role in the transmembrane transport of a wide range of chemotherapeutic agents. Polymorphisms in these genes can affect the efficiency of drug uptake and efflux in target tissues including the Liver, kidneys, and bone marrow there by altering systemic drug exposure and toxicity profiles. A notable example is the ABCB1 C3435T polymorphism, which has been associated with an elevated risk of doxorubicin induced neutropenia, likely due to its influence on glycoprotein expression and drug efflux capacity (Hao et al., 2020; Megías-Vericat et al., 2022).

In summary, genetic polymorphisms in drug metabolizing enzymes and transporter genes significantly influence individual susceptibility to chemotherapy-related bone marrow toxicity. This area has become a critical focus in the development of precision oncology and risk stratification strategies for chemotherapy. While genotype guided chemotherapy dosing is not yet widely adopted across all cancer types, growing evidence from recent meta-analyses suggests that individualized dose adjustment based on genetic profiles holds significant clinical promise, particularly for high risk drugs patient populations.

## 5 Limitations

Several limitations should be noted in this study. First, substantial heterogeneity was observed among the included studies, likely arising from differences in methodological design, study duration, demographic characteristics, geographic regions, healthcare systems, and other unmeasured confounding variables, which may limit the interpretability and generalizability of the findings. Second, the analysis included only studies published in English and Chinese, which may have introduced language bias and excluded relevant literature in other languages. This limitation could reduce the comprehensiveness and global representativeness of the results. Moreover, potential constraints in database selection, search strategy, and indexing may have contributed to incomplete retrieval of eligible studies. Third, publication bias cannot be ruled out, as studies with negative or nonsignificant findings may be underrepresented, potentially leading to an overestimation of the pooled effect sizes. In addition, variations in methodological quality, sample size, and reporting standards among the included studies could have influenced the robustness of our results. Most of the included studies were rated as having moderate quality, which may weaken the overall strength of evidence. Finally, most studies were conducted in Asian populations, which may limit the generalizability of our conclusions to non Asian or global populations.

## 6 Conclusion

In conclusion, this meta-analysis found that CIM occurred in nearly half (47.9%) of BC patients, underscoring the need for greater clinical vigilance. Older age, low BMI, advanced disease stage, diabetes, and impaired Liver function were identified as major risk factors. These factors may guide risk stratification and inform closer hematologic monitoring, prophylactic G-CSF use, and individualized chemotherapy dosing. Taxane- and anthracycline-based combination regimens, particularly TAC, were associated with the highest risk of CIM, highlighting the importance of proactive prevention and tailored treatment strategies. Future studies should focus on developing validated predictive models and conducting prospective research to confirm causal associations and optimize CIM prevention and management in BC patients.
