# Supplementary material for: The risk factors for chemotherapy myelosuppression in breast cancer: a systematic review and meta-analysis
Source: Front Genet. 2025 Dec 8;16:1704489. doi: 10.3389/fgene.2025.1704489 (PMC12719511; doi:10.3389/fgene.2025.1704489)
Supplement: Supplementary file 1 [file Supplementaryfile1.docx]

Pubmed search formulation

| Search | Query | Results |
| --- | --- | --- |
| #1 | Search: "Breast Neoplasms"[Mesh] | 364,731 |
| #2 | Search: **"**Breast Tumor"[Title] OR "Breast Cancer"[Title] OR "Breast Malignant Tumor"[Title] OR "Mammary Cancer "[Title] OR "Breast Carcinoma"[Title] OR "Breast Carcinomas"[Title] | 253,423 |
| #3 | Search: "Leukopenia"[Mesh] | 41,358 |
| #4 | Search: "Neutropenia"[Mesh] | 21,118 |
| #5 | Search: "Anemia"[Mesh] | 181,836 |
| #6 | Search: "Thrombocytopenia"[Mesh] | 55,617 |
| #7 | Search: ((("Leukopenia"[Mesh]) OR ("Neutropenia"[Mesh])) OR ("Anemia"[Mesh])) OR ("Thrombocytopenia"[Mesh]) Sort by: Most Recent | 259,897 |
| #8 | Search: "Bone marrow suppression"[Title] OR " severe bone marrow suppression"[Title] OR " leukopenia"[Title] OR " neutropenia"[Title] OR " anemia"[Title] OR " thrombocytopenia"[Title] Sort by: Most Recent | 80,348 |
| #9 | Search: (((("Leukopenia"[Mesh]) OR ("Neutropenia"[Mesh])) OR ("Anemia"[Mesh])) OR ("Thrombocytopenia"[Mesh])) OR ("Bone marrow suppression"[Title] OR " severe bone marrow suppression"[Title] OR " leukopenia"[Title] OR " neutropenia"[Title] OR " anemia"[Title] OR " thrombocytopenia"[Title]) | 271,238 |
| #10 | Search: "Risk Factors"[Mesh] | 1,024,514 |
| #11 | Search: "risk factor*"[Title] OR " risk variable*"[Title] OR " predictor*"[Title] OR " prediction"[Title] OR " predict factor*"[Title] OR " prognostic factor*"[Title] OR " epidemiologic factor*"[Title] OR " epidemiologic variable*"[Title] OR " relevant factor*"[Title] OR " related variable*"[Title] OR " contributing factor*"[Title] | 426,946 |
| #12 | Search: ("Risk Factors"[Mesh]) OR ("risk factor*"[Title] OR " risk variable*"[Title] OR " predictor*"[Title] OR " prediction"[Title] OR " predict factor*"[Title] OR " prognostic factor*"[Title] OR " epidemiologic factor*"[Title] OR " epidemiologic variable*"[Title] OR " relevant factor*"[Title] OR " related variable*"[Title] OR " contributing factor*"[Title]) | 1,289,094 |
| #13 | Search: (("Breast Tumor"[Title] OR "Breast Cancer"[Title] OR "Breast Malignant Tumor"[Title] OR "Mammary Cancer "[Title] OR "Breast Carcinoma"[Title] OR "Breast Carcinomas"[Title]) AND ((((("Leukopenia"[Mesh]) OR ("Neutropenia"[Mesh])) OR ("Anemia"[Mesh])) OR ("Thrombocytopenia"[Mesh])) OR ("Bone marrow suppression"[Title] OR " severe bone marrow suppression"[Title] OR " leukopenia"[Title] OR " neutropenia"[Title] OR " anemia"[Title] OR " thrombocytopenia"[Title]))) AND (("Risk Factors"[Mesh]) OR ("risk factor*"[Title] OR " risk variable*"[Title] OR " predictor*"[Title] OR " prediction"[Title] OR " predict factor*"[Title] OR " prognostic factor*"[Title] OR " epidemiologic factor*"[Title] OR " epidemiologic variable*"[Title] OR " relevant factor*"[Title] OR " related variable*"[Title] OR " contributing factor*"[Title])) | 98 |

Cochrane Library search formulation

| Search | Query | Results |
| --- | --- | --- |
| #1 | MeSH descriptor: [Breast Neoplasms] explode all trees | 20,476 |
| #2 | (Breast Tumor):ti,ab,kw OR (Breast Cancer):ti,ab,kw OR (Breast Malignant Tumor):ti,ab,kw OR (Mammary Cancer ):ti,ab,kw OR (Breast Carcinoma):ti,ab,kw OR (Breast Carcinomas):ti,ab,kw | 48,545 |
| #3 | #1OR #2 | 49,424 |
| #4 | MeSH descriptor: [Leukopenia] explode all trees | 3,130 |
| #5 | MeSH descriptor: [Neutropenia] explode all trees | 2,230 |
| #6 | MeSH descriptor: [Anemia] explode all trees | 7,313 |
| #7 | MeSH descriptor: [Thrombocytopenia] explode all trees | 1,808 |
| #8 | #4 OR #5 OR #6 OR #7 | 11,537 |
| #9 | (Bone marrow suppression):ti,ab,kw OR (severe bone marrow suppression):ti,ab,kw OR (leukopenia):ti,ab,kw OR (neutropenia):ti,ab,kw OR (anemia):ti,ab,kw OR (thrombocytopenia):ti,ab,kw | 44,318 |
| #10 | #8 OR #9 | 45,410 |
| #11 | MeSH descriptor: [Risk Factors] explode all trees | 37,676 |
| #12 | (risk factor*):ti,ab,kw OR (risk variable*):ti,ab,kw OR (predictor*):ti,ab,kw OR (prediction):ti,ab,kw OR (predict factor*):ti,ab,kw OR (prognostic factor*):ti,ab,kw OR (epidemiologic factor*):ti,ab,kw OR (epidemiologic variable*):ti,ab,kw OR (relevant factor*):ti,ab,kw OR (related variable*):ti,ab,kw OR (contributing factor*):ti,ab,kw | 209,510 |
| #13 | #11 OR #12 | 209,513 |
| #14 | #3 AND #10 AND #13 | 512 |

Embase search formulation

| Search | Query | Results |
| --- | --- | --- |
| #1 | ‘breast tumor'/exp | 737,952 |
| #2 | ‘breast neoplasms':ti OR ‘breast cancer':ti OR ‘breast malignant tumor':ti OR ‘mammary cancer':ti OR 'breast carcinoma':ti OR ‘breast carcinomas':ti | 357,110 |
| #3 | #1 OR #2 | 750,010 |
| #4 | ‘bone marrow suppression'/exp | 34,644 |
| #5 | ‘leukopenia'/exp | 273,424 |
| #6 | ‘neutropenia'/exp | 157,658 |
| #7 | ‘anemia'/exp | 541,992 |
| #8 | ‘thrombocytopenia'/exp | 251,011 |
| #9 | #4 OR #5 OR #6 OR #7 OR #8 | 851,268 |
| #10 | ‘bone marrow suppression':ti OR ‘severe bone marrow suppression':ti OR ‘leukopenia':ti OR ‘neutropenia':ti OR ‘anemia':ti OR‘thrombocytopenia':ti | 106,929 |
| #11 | #9 OR #10 | 855,134 |
| #12 | ‘risk factor'/exp | 1,513,289 |
| #13 | ‘risk factor*':ti OR ‘risk variable*':ti OR 'predictor*':ti OR ‘prediction':ti OR ‘predict factor*':ti OR 'prognostic factor*':ti OR ‘epidemiologic factor*':ti OR ‘epidemiologic variable*':ti OR ’‘relevant factor*':ti OR ‘related variable*':ti OR ‘contributing factor*':ti | 590,332 |
| #14 | #12 OR #13 | 1,864,361 |
| #15 | #3 AND #11 AND #14 | 1,391 |

Web of science search formulation

| Search | Query | Results |
| --- | --- | --- |
| #1 | ((((((TS=(Breast Neoplasms)) OR TS=(Breast Tumor)) OR TS=(Breast Cancer)) OR TS=(Breast Malignant Tumor)) OR TS=(Mammary Cancer )) OR TS=(Breast Carcinoma)) OR TS=(Breast Carcinomas) | 984,719 |
| #2 | (((((TS=(Bone marrow suppression)) OR TS=(severe bone marrow suppression)) OR TS=(leukopenia)) OR TS=(neutropenia)) OR TS=(anemia)) OR TS=(thrombocytopenia) | 491,903 |
| #3 | (((((((((((TS=(risk factors)) OR TS=(risk factor*)) OR TS=(risk variable*)) OR TS=(predictor*)) OR TS=(prediction)) OR TS=(predict factor*)) OR TS=(prognostic factor*)) OR TS=(epidemiologic factor*)) OR TS=(epidemiologic variable*)) OR TS=(relevant factor*)) OR TS=(related variable*)) OR TS=(contributing factor*) | 6,790,699 |
| #4 | #1 AND #2 AND #3 | 2,820 |
| #5 | ((((((TI=(Breast Neoplasms)) OR TI=(Breast Tumor)) OR TI=(Breast Cancer)) OR TI=(Breast Malignant Tumor)) OR TI=(Mammary Cancer )) OR TI=(Breast Carcinoma)) | 424,730 |
| #6 | (((((TI=(Bone marrow suppression)) OR TI=(severe bone marrow suppression)) OR TI=(leukopenia)) OR TI=(neutropenia)) OR TI=(anemia)) OR TI=(thrombocytopenia) | 127,417 |
| #7 | (((((((((((TI=(risk factors)) OR TI=(risk factor*)) OR TI=(risk variable*)) OR TI=(predictor*)) OR TI=(prediction)) OR TI=(predict factor*)) OR TI=(prognostic factor*)) OR TI=(epidemiologic factor*)) OR TI=(epidemiologic variable*)) OR TI=(relevant factor*)) OR TI=(related variable*)) OR TI=(contributing factor*) | 828,313 |
| #8 | #5 AND #6 AND #7 | 41 |

Medline search formulation search formulation

| Search | Query | Results |
| --- | --- | --- |
| #1 | Breast Neoplasms (MeSH) | 350,572 |
| #2 | ((((((TI=(Breast Neoplasms)) OR TI=(Breast Tumor)) OR TI=(Breast Cancer)) OR TI=(Breast Malignant Tumor)) OR TI=(Mammary Cancer )) OR TI=(Breast Carcinoma)) OR TI=(Breast Carcinomas) | 286,273 |
| #3 | #1 OR #2 | 413,860 |
| #4 | MH=(leukopenia) | 8,785 |
| #5 | MH=(Bone marrow suppression) | 0 |
| #6 | MH=(neutropenia) | 19,442 |
| #7 | MH=(anemia) | 56,879 |
| #8 | MH=(thrombocytopenia) | 31,436 |
| #9 | #4 OR #6 OR #7 OR #8 | 108,942 |
| #10 | (((((TI=(Bone marrow suppression)) OR TI=(severe bone marrow suppression)) OR TI=(leukopenia)) OR TI=(neutropenia)) OR TI=(anemia)) OR TI=(thrombocytopenia) | 98,265 |
| #11 | #9 OR #10 | 160,294 |
| #12 | MH=(risk factors) | 1,019,383 |
| #13 | (((((((((((TI=(risk factors)) OR TI=(risk factor*)) OR TI=(risk variable*)) OR TI=(predictor*)) OR TI=(prediction)) OR TI=(predict factor*)) OR TI=(prognostic factor*)) OR TI=(epidemiologic factor*)) OR TI=(epidemiologic variable*)) OR TI=(relevant factor*)) OR TI=(related variable*)) OR TI=(contributing factor*) | 466,243 |
| #14 | #3 AND #11 AND #13 | 53 |

Scopus search formulation

| Search | Query | Results |
| --- | --- | --- |
| #1 | ( ABS ( breast AND neoplasms ) OR ABS ( breast AND tumor ) OR ABS ( breast AND cancer )  OR ABS ( mammary AND cancer ) OR ABS ( breast AND carcinoma ) OR ABS ( breast AND carcinomas ) ) | 268,685 |
| #2 | (ABS ( bone AND marrow AND suppression ) OR ABS ( severe AND bone AND marrow AND  suppression )OR ABS ( leukopenia ) OR ABS ( neutropenia ) OR ABS ( anemia ) OR ABS ( thrombocytopenia ) ) | 504,446 |
| #3 | ( ABS ( risk AND factors ) OR ABS ( risk AND factor* ) OR ABS ( risk AND variable* ) OR ABS ( predictor* ) OR ABS ( prediction ) OR ABS ( predict AND factor* ) OR ABS ( prognostic AND factor* ) OR ABS ( epidemiologic AND factor* ) OR ABS ( epidemiologic AND variable* ) OR ABS ( relevant AND factor* ) OR ABS ( related AND variable* ) OR ABS ( contributing AND factor* ) ) | 4,943,965 |
| #4 | #1 AND #2 AND #3 | 925 |

知网检索式

| 步骤 | 检索式 | 结果 |
| --- | --- | --- |
| #1 | (主题:乳腺癌)OR(主题:乳腺恶性肿瘤)OR(主题:乳肿块)OR(主题:乳房癌)AND(主题:骨髓抑制)OR(主题:重度骨髓抑制)OR(主题:白细胞减少)OR(主题:中性粒细胞减少)OR(主题:贫血)OR(主题:血小板减少)AND(主题:风险因素)OR(主题:预测因素)OR(主乳腺癌忌题:预测变量)OR(主题:影响因素)OR(主题:相关因素)OR(主题:危及影响区险因素) | 78 |

CBM search formulation

| 步骤 | 检索式 | 结果 |
| --- | --- | --- |
| #1 | "乳腺肿瘤"[不加权:扩展] | 462,011 |
| #2 | "乳腺癌"[摘要:智能] OR "乳腺恶性肿瘤"[摘要:智能] OR "乳腺肿块"[摘要:智能] OR "乳房癌"[常用字段:智能] | 447,993 |
| #3 | (("乳腺肿瘤"[不加权:扩展]) OR (("乳腺癌"[摘要:智能] OR "乳腺恶性肿瘤"[摘要:智能] OR "乳腺肿块"[摘要:智能] OR "乳房癌"[常用字段:智能]))) | 607,285 |
| #4 | "白细胞减少"[不加权:扩展] | 45,353 |
| #5 | "中性粒细胞减少"[不加权:扩展] | 19,864 |
| #6 | "贫血"[不加权:扩展] | 221,059 |
| #7 | "血小板减少"[不加权:扩展] | 71,800 |
| #8 | ("血小板减少"[不加权:扩展]) OR ("贫血"[不加权:扩展]) OR ("中性粒细胞减少"[不加权:扩展]) OR ("白细胞减少"[不加权:扩展]) | 319,366 |
| #9 | "骨髓抑制"[摘要:智能] OR "重度骨髓抑制"[摘要:智能] OR "白细胞减少"[摘要:智能] OR "中性粒细胞减少"[摘要:智能] OR "贫血"[摘要:智能] OR "血小板减少"[摘要:智能] | 281,404 |
| #10 | (((("血小板减少"[不加权:扩展]) OR ("贫血"[不加权:扩展]) OR ("中性粒细胞减少"[不加权:扩展]) OR ("白细胞减少"[不加权:扩展]))) OR (("骨髓抑制"[摘要:智能] OR "重度骨髓抑制"[摘要:智能] OR "白细胞减少"[摘要:智能] OR "中性粒细胞减少"[摘要:智能] OR "贫血"[摘要:智能] OR "血小板减少"[摘要:智能]))) | 483,389 |
| #11 | "危险因素"[不加权:扩展] | 1,113,181 |
| #12 | "风险因素"[摘要:智能] OR "预测因素"[摘要:智能] OR "预测变量"[摘要:智能] OR "影响因素"[摘要:智能] OR "相关因素"[摘要:智能] | 332,075 |
| #13 | (("危险因素"[不加权:扩展]) OR (("风险因素"[摘要:智能] OR "预测因素"[摘要:智能] OR "预测变量"[摘要:智能] OR "影响因素"[摘要:智能] OR "相关因素"[摘要:智能]))) | 1,411,852 |
| #14 | ((((((("乳腺肿瘤"[不加权:扩展]) OR (("乳腺癌"[摘要:智能] OR "乳腺恶性肿瘤"[摘要:智能] OR "乳腺肿块"[摘要:智能] OR "乳房癌"[常用字段:智能]))))) AND (((((("血小板减少"[不加权:扩展]) OR ("贫血"[不加权:扩展]) OR ("中性粒细胞减少"[不加权:扩展]) OR ("白细胞减少"[不加权:扩展]))) OR (("骨髓抑制"[摘要:智能] OR "重度骨髓抑制"[摘要:智能] OR "白细胞减少"[摘要:智能] OR "中性粒细胞减少"[摘要:智能] OR "贫血"[摘要:智能] OR "血小板减少"[摘要:智能]))))))) AND (((("危险因素"[不加权:扩展]) OR (("风险因素"[摘要:智能] OR "预测因素"[摘要:智能] OR "预测变量"[摘要:智能] OR "影响因素"[摘要:智能] OR "相关因素"[摘要:智能])))))) | 258 |

万方数据库

| 步骤 | 检索式 | 结果 |
| --- | --- | --- |
| #1 | 主题:(乳腺癌 OR 乳腺恶性肿瘤 OR 乳腺肿块 OR 乳房癌) and 主题:(骨髓抑制 OR 重度骨髓抑制 OR 白细胞减少 OR 中性粒细胞减少 OR 贫血 OR 血小板减少) and 主题:(风险因素 OR 预测因素 OR 预测变量 OR 影响因素 OR 相关因素 OR 危险因素 | 380 |

维普检索式

| 检索式 | 检索式 | 结果 |
| --- | --- | --- |
| #1 | (((((题名=乳腺癌 OR 题名=乳腺恶性肿瘤) OR 题名=乳腺肿块) OR 题名=乳房癌) AND (((((题名=骨髓抑制 OR 题名=重度骨髓抑制) OR 题名=白细胞减少) OR 题名=中性粒细胞减少) OR 题名=贫血) OR 题名=血小板减少)) AND (((((题名=风险因素 OR 题名=预测因素) OR 题名=预测变量) OR 题名=影响因素) OR 题名=相关因素) OR 题名=危险因素)) | 15 |
| #2 | (((((关键词=乳腺癌 OR 关键词=乳腺恶性肿瘤) OR 关键词=乳腺肿块) OR 关键词=乳房癌) AND (((((关键词=骨髓抑制 OR 关键词=重度骨髓抑制) OR 关键词=白细胞减少) OR 关键词=中性粒细胞减少) OR 关键词=贫血) OR 关键词=血小板减少)) AND ((((关键词=风险因素 OR 关键词=预测因素) OR 关键词=预测变量) OR 关键词=影响因素) OR 关键词=相关因素OR 危险因素)) | 9 |
| #3 | (((((题名或关键词=乳腺癌 OR 题名或关键词=乳腺恶性肿瘤) OR 题名或关键词=乳腺肿块) OR 题名或关键词=乳房癌) AND (((((题名或关键词=骨髓抑制 OR 题名或关键词=重度骨髓抑制) OR 题名或关键词=白细胞减少) OR 题名或关键词=中性粒细胞减少) OR 题名或关键词=贫血) OR 题名或关键词=血小板减少)) AND ((((题名或关键词=风险因素 OR 题名或关键词=预测因素) OR 题名或关键词=预测变量) OR 题名或关键词=影响因素) OR 题名或关键词=相关因素OR 危险因素)) | 19 |
| #4 | (((((摘要=乳腺癌 OR 摘要=乳腺恶性肿瘤) OR 摘要=乳腺肿块) OR 摘要=乳房癌) AND (((((摘要=骨髓抑制 OR 摘要=重度骨髓抑制) OR 摘要=白细胞减少) OR 摘要=中性粒细胞减少) OR 摘要=贫血) OR 摘要=血小板减少)) AND ((((摘要=风险因素 OR 摘要=预测因素) OR 摘要=预测变量) OR 摘要=影响因素) OR 摘要=相关因素OR 危险因素)) | 151 |
